# A Case of Leukemia Cutis with Acute Myeloid Leukemia on Azacitidine Therapy

**DOI:** 10.4274/tjh.2016.0220

**Published:** 2017-06-01

**Authors:** Asude Kara, Aslı Akın Belli, Volkan Karakuş, Yelda Dere, Erdal Kurtoğlu

**Affiliations:** 1 Department of Dermatology, Muğla Sıtkı Koçman University Training and Research Hospital, Muğla, Turkey; 2 Department of Hematology, Muğla Sıtkı Koçman University Training and Research Hospital, Muğla, Turkey; 3 Department of Pathology, Muğla Sıtkı Koçman University Faculty of Medicine, Mugla, Turkey; 4 Department of Hematology, Antalya Training and Research Hospital, Antalya, Turkey

**Keywords:** Acute myeloid leukemia, Azacitidine, Leukemia cutis

## To The Editor,

Leukemia cutis (LC) is an extramedullary form of leukemia. The frequency and onset age of LC depends on the subtype of underlying leukemia. Clinical presentations of LC can be variable but it generally appears as nodules and plaques [1]. Herein, we report a case of LC with acute myeloid leukemia on azacitidine therapy.

A 70-year-old male presented with spontaneous ecchymoses and weakness in the Hematology Outpatient Clinic. Physical examination was normal. Laboratory tests were as follows: hemoglobin: 4.1 g/dL, leukocyte count: 17.600/mm^3^, platelet count: 57.000/mm^3^,, and lactate dehydrogenase: 605 U/L. Peripheral blood smear revealed 60% myeloblasts. Eighty percent of blastic infiltration and positive staining with myeloperoxidase (MPO) were detected in the bone marrow biopsy. The patient was diagnosed with acute myelomonocytic leukemia (AML-M4) with the morphological and immunopathological findings. Flow cytometry of the bone marrow or peripheral blood was not done. In the conventional cytogenetic analysis done before the treatment, 20 metaphases were detected. Six of them had “add” (46,XY,add(8)(q24)[6]) and 10 were diploids and 4 were hypodiploids. In the fluorescence in situ hybridization (FISH) analysis [5q31, t(15;17)(q22-24;q21), trisomy 8, t(9;22)(q34;q11.2), inv(16)(p13q22), del7q31, del/inv11q23, monosomy 7, t(8;21)(q21.3;q22), and del20q], there was no abnormality.

**Figure 1:**
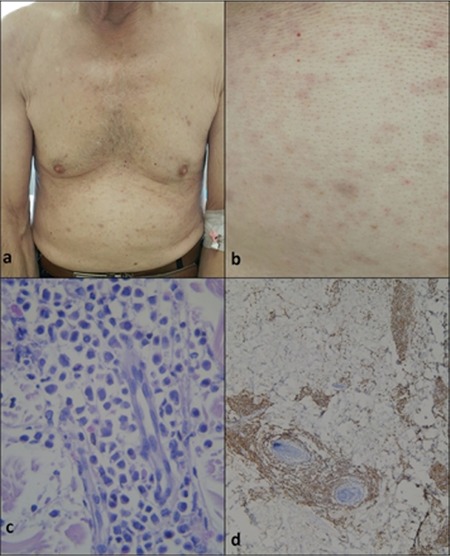
a) Multiple discrete, erythematous papules and nodules on the trunk. b) Closer view of the erythematous papules on the abdomen. c) Blastic cells with pleomorphic nuclei and narrow cytoplasm in the dermis (hematoxylin & eosin, original magnification 400^x^). d) Positive staining of the blasts with myeloperoxidase (original magnification 40^x^).

After four cycles of therapy with azacitidine (75 mg/m2 daily for 7 days in a month), the need for red blood cells decreased but the need for platelets remained. Furthermore, some skin lesions appeared on the trunk. On dermatological examination, multiple discrete, violaceous-erythematous papules and nodules were observed on the trunk (Figures 1a and 1b). Histopathological examination of the skin lesions showed blastic cell infiltration with large pleomorphic nuclei and narrow cytoplasm in the dermis and also positive staining with CD34, CD117, and MPO (Figures 1c and 1d). The patient was diagnosed with LC with AML-M4 relapsed in the bone marrow synchronously and the therapy regimen was changed from azacitidine to cytosine-arabinoside. The same FISH panel was obtained.

LC is a rare disease characterized by leukemic cell infiltration in the dermis, subcutaneous tissue, and blood vessels. The frequency of LC is about 2%-3% in patients with AML [2]. LC mimics various dermatoses. Histopathological analysis of the lesions has an important role in the diagnosis [3]. There is no special treatment for LC and the treatment of the underlying leukemia generally improves skin lesions. Unfortunately, skin involvement of leukemia indicates poor prognosis [3].

Azacitidine is an approved and well-tolerated drug in the treatment of elderly AML patients in particular [4]. Recently, we noticed that LC cases have been reported in patients with chronic myeloid leukemia and myelodysplastic syndrome on azacitidine therapy [5]. Similarly, azacitidine therapy at the current dosage may have been insufficient in our patient and thus the cutaneous involvement developed. We want to emphasize that patients developing LC on azacitidine therapy should be accepted as refractory to the therapy and salvage therapy should be planned.
